# Paediatric obesity: a systematic review and pathway mapping of metabolic alterations underlying early disease processes

**DOI:** 10.1186/s10020-021-00394-0

**Published:** 2021-11-06

**Authors:** Margot De Spiegeleer, Ellen De Paepe, Lieven Van Meulebroek, Inge Gies, Jean De Schepper, Lynn Vanhaecke

**Affiliations:** 1 Laboratory of Chemical Analysis, Department of Translational Physiology, Infectiology and Public Health, Ghent University, Salisburylaan 133, 9820 Merelbeke, Belgium; 2 KidZ Health Castle, Universitair Ziekenhuis Brussel, Vrije Universiteit Brussel, Laarbeeklaan 101, 1090 Brussel, Belgium; 3 Department of Internal Medicine and Pediatrics, Faculty of Medicine and Health Sciences, Ghent University, Corneel Heymanslaan 10, 9000 Ghent, Belgium; 4 Institute for Global Food Security, School of Biological Sciences, Queen’s University, University Road, Belfast, BT7 1NN UK

**Keywords:** Metabolomics, Lipidomics, Childhood obesity, Metabolic disease, Diabetes, Impaired glucose tolerance

## Abstract

**Background:**

The alarming trend of paediatric obesity deserves our greatest awareness to hinder the early onset of metabolic complications impacting growth and functionality. Presently, insight into molecular mechanisms of childhood obesity and associated metabolic comorbidities is limited.

**Main body of the abstract:**

This systematic review aimed at scrutinising what has been reported on putative metabolites distinctive for metabolic abnormalities manifesting at young age by searching three literature databases (Web of Science, Pubmed and EMBASE) during the last 6 years (January 2015–January 2021). Global metabolomic profiling of paediatric obesity was performed (multiple biological matrices: blood, urine, saliva and adipose tissue) to enable overarching pathway analysis and network mapping. Among 2792 screened Q1 articles, 40 met the eligibility criteria and were included to build a database on metabolite markers involved in the spectrum of childhood obesity. Differential alterations in multiple pathways linked to lipid, carbohydrate and amino acid metabolisms were observed. High levels of lactate, pyruvate, alanine and acetate marked a pronounced shift towards hypoxic conditions in children with obesity, and, together with distinct alterations in lipid metabolism, pointed towards dysbiosis and immunometabolism occurring early in life. Additionally, aberrant levels of several amino acids, most notably belonging to tryptophan metabolism including the kynurenine pathway and its relation to histidine, phenylalanine and purine metabolism were displayed. Moreover, branched-chain amino acids were linked to lipid, carbohydrate, amino acid and microbial metabolism, inferring a key role in obesity-associated insulin resistance.

**Conclusions:**

This systematic review revealed that the main metabolites at the crossroad of dysregulated metabolic pathways underlying childhood obesity could be tracked down to one central disturbance, i.e. impending insulin resistance for which reference values and standardised measures still are lacking. In essence, glycolytic metabolism was evinced as driving energy source, coupled to impaired Krebs cycle flux and ß-oxidation. Applying metabolomics enabled to retrieve distinct metabolite alterations in childhood obesity(-related insulin resistance) and associated pathways at early age and thus could provide a timely indication of risk by elucidating early-stage biomarkers as hallmarks of future metabolically unhealthy phenotypes.

**Supplementary Information:**

The online version contains supplementary material available at 10.1186/s10020-021-00394-0.

## Introduction

At present, millions of children, of whom even under the age of 5, are afflicted with overweight and obesity (WHO 2020), rendering the globesity pandemic one of the utmost health concerns of the twenty-first century. As there prevails a strong trend of childhood obesity tracking into adulthood in an estimated 80% of cases (Simmonds et al. 2016), the unabated rise of paediatric obesity might anticipate the onset of impaired fasting glucose (IFG) and impaired glucose tolerance (IGT) to teen age and full-blown diabetes type 2 to early adulthood (Haemer et al. 2014; Candler et al. 2018). In addition, IFG, IGT and insulin resistance (IR) have been appointed high-risk states introducing the metabolically unhealthy sequelae of obesity including dyslipidemia, hypertension, metabolic syndrome and atherosclerosis which frequently progress into diabetes type 2 but also cardiovascular disease and cancer (WHO 2018; Cobb et al. 2016). It is however worth pointing out that metabolic processes initiating the micro- and macrovascular events are likely to occur already in childhood, predisposing children to the development and accelerated onset of several long-term complications (Wideman et al. 2013; Tuomi et al. 2014; Weiss et al. 2005). Therefore, focus should shift from treatment of disease towards risk-stratification at a crucial stage in life, i.e. childhood, during which metabolic impairments are still reversible.

IR is a forerunner state in metabolic disease onset since it already rises years before pubertal onset (Weiss et al. 2005; Tsay et al. 2010). Consequently, IR is more strongly associated with a metabolically unhealthy status than is obesity, leading to the use of insulin sensitivity indices (Valeria et al. 2015). The gold standard ‘the hyperinsulinemic clamps’ for directly assessing IR is time consuming, labor intensive, and overall expensive (Tam et al. 2012). Therefore, several alternate measures have been developed amongst which the homeostatic model assessment of IR (HOMA-IR), the quantitative insulin sensitivity index (QUICKI) and the Matsuda index. The HOMA-IR and QUICKI serve as a primary reflection of hepatic insulin sensitivity, whereas the Matsuda index includes both skeletal muscle and hepatic IR, yet inferring the need of an oral glucose tolerance test (OGTT) which is unpopular with primary care physicians and patients (Cobb et al. 2016). Recently, simpler and more cost-effective clinical surrogate markers for IR have been introduced, e.g. the triglyceride glucose index (Hong et al. 2020). In clinical practice however, these measures are not yet executed as a standard methodology since no consensus on internationally agreed reference value(s) for (ab)normal insulin sensitivity in children exist (Haemer et al. 2014). Thus, apart from an adjusted body mass index (BMI) in diagnosing obesity in children 2 years and older (Cuda and Censani 2019), it still remains unclear when to screen for metabolic complications and assessing (ab)normal insulin sensitivity during childhood (American Diabetes Association 2018). Hence, an urging quest for diagnosis of IR and for prognosis and/or prediction regarding the early onset of associated metabolic disease in children is markedly present. Hereto, metabolite patterns offer a valid tool to profile and stratify high risk obese and/or IR states (Cobb et al. 2016).

Today, two pandemics meet, in which it has been evinced that obesity and its highly associated metabolic traits are strongly related to high risk of morbidity and mortality (Zhao et al. 2016) and, at present, are independently associated with greater susceptibility and adverse outcomes of COVID-19 (Lockhart and O’Rahilly 2020). It is worth pointing out that metabolomics has stepped forward as a valid alternate analysis technology in diverse diseases including obesity as well as COVID-19, i.e. the detection of characteristic molecular changes in the biofluids of patients (Shen et al. 2020).

The close relatedness of metabolites to one’s phenotype and the analytical advances of metabolomics in rapid and high-throughput technologies are key-drivers to gain insights into the pathophysiology of a diverse spectrum of diseases (Spiegeleer et al. 2020). Diet and lifestyle, genetic predisposition and diverse environmental contributors shape the obesogenic landscape from early age on and as such, inevitably correlate to the current emergence of paediatric obesity. Moreover, the complex synergistic intertwining of such influencing variables is vital (Snijders et al. 2016). In this context, metabolomics reveals a far more comprehensive metabolic signature than clinical blood analyses reluctantly performed in young children. Also, blood metabolites are dependent on systemic appearance rendering it rather difficult to trace metabolic aberrations to the respective organ’s physiology and its specific cause (Adams 2011). Therefore, implementing a multi-matrix approach for disease-related biomarker detection or pathway elucidation offers the potential to expose pathophysiological dynamicity, e.g. unravelling the interplay between the host, diet and microbiome (Spiegeleer et al. 2020; Paepe et al. 2018).

The present global eminence of childhood obesity and associated early onset of asymptomatic complications, has not escaped the awareness of metabolomics research. In 2016, Zhao et al. (2016) reviewed IR in childhood obesity on the basis of blood metabolomics studies. Metabolisms of amino acids and lipids were most affected. In specific, branched-chain (BCAAs) and aromatic amino acids (AAAs) as well as acylcarnitines were appointed as closely related to IR and future metabolic risk. The aims of this systematic review were to further highlight and consolidate the importance of recent metabolomics studies applied on various types of biofluids and to include pathway mapping. Thereby, subsequent to an extensive literature search and database construction, pathway analysis was performed by MetaboAnalyst 5.0 and MetScape 3 in providing an overarching metabolomics signature of childhood obesity. The significant metabolites that were extracted from included studies were reviewed in light of their potential value as early-stage hallmarks of future metabolically unhealthy phenotypes together with in-depth interpretation of disturbances observed in their associated metabolic pathways.

## Materials/subjects and methods

### Literature search strategy and quality scoring

Published literature in English (from January 2015 up to January 2021) was searched to identify case–control or cohort studies in three electronic databases, i.e. Web of Science, Pubmed and EMBASE. The pragmatic search methodology and terminology was effectuated according to the PICO framework and PRISMA statement (Liberati et al. 2009) (Additional file 1: Table S1). Selection of retrieved studies and respective quality assessment was effectuated independently (author MDS) and in duplicate (author LVM) based on the Newcastle–Ottawa Quality Assessment scale (Bae 2016), and adjusted specifically for case reports and case series where necessary (Murad et al. 2018) (Additional file 1: Tables S2 and S3). The study protocol was registered in the PROSPERO registry (ID 181149).

### Study selection: in- and exclusion criteria

Articles were selected based on pre-defined in- and exclusion criteria (Table 1). Additional articles were included by reference tracking as secondary source. The unique titles and abstracts resulting from the search strategy which focused on Q1 papers and concurrent duplicate removal were reviewed independently by two authors (MDS and LVM). The full text paper was assessed correspondingly, by the same two authors (MDS and LVM) and a third independent reviewer (LV) resolved discrepancies regarding eligibility.

**Table 1:** Characteristics of the included studies

	Study attributes/characteristics of groups studied
Inclusion criteria	
Study design	Population-based observational studies and (experiment) trials: case–control, cohort and case-series
Population/age	Children and/or adolescents (0–19 years^a^)
Outcome	Obesity and/or related metabolic abnormalities (impaired glucose tolerance, insulin resistance, prediabetes, diabetes mellitus type 2, metabolic syndrome) in metabolomics research
Biofluid	Blood (serum and plasma), urine, excretion, faeces, saliva, tissue, hair, nails
Diagnostics	Clinician-based: BMI diagnostic criteria, oral glucose tolerance test, parameters/criteria for metabolic syndrome, cardiovascular disease risk, non-alcoholic fatty liver disease, etc
Exclusion criteria	
Language	Language other than English
Document type	Other than article (e.g. review, letters, conference abstracts)
Age/disease	Study population of adults only and in utero/maternal studies (e.g. gestational diabetes), participant population with any thyroid or metabolic disorder under treatment (e.g. diabetes, cardiovascular disease)
Type of study	Exposure study, genomics, transcriptomics, proteomics, microbiomics and intervention studies

A detailed description of the in- and exclusion criteria on the basis of which articles were retrieved and selected articles were withheld for final assessment

^a^Based on WHO definition

Both cross-sectional and longitudinal study designs were considered eligible if those were human studies and not exclusively focused on interventions including therapeutics, diet and surgery to mainstream the study population and abolish confounding factors regarding metabolome perturbations. Furthermore, as a quality requirement, the classification of obesity had to be clearly addressed and based on any local or international reference values in order to enable the comparison of childhood obesity across various ages. Both articles using metabolic profiling and fingerprinting through state-of-the-art high-throughput metabolomics techniques in a wide range of biological matrices were included (Table 1). To limit the extensive coverage to metabolomics studies, other omics investigations that merely targeted genomics, transcriptomics, proteomics or microbiomics were excluded as was the case for prevalence studies.

### Data extraction and variable listing: the construction of a childhood obesity metabolomics database

From each included study, targeted metabolites (identification tier 1 and 2) that were significantly altered in the group of children with overweight or obesity, i.e. compared to a healthy weight control group and in correlation with specific descriptors, were selected from tables, figures and supplemental data. Extracted items included qualitative data: metabolite characteristics, i.e. name and class (according to the Human Metabolome Database (Wishart et al. 2018) or LIPID MAPS (Fahy et al. 2008) classification, ChEBI and KEGG identification, chemical formula, identification tier, the biological matrix studied and the analytical measurement technique applied. Also, the study design addressed and population characteristics, e.g. age, BMI *z*-score, percentage of female participants, etc., and if available quantitative information regarding outcome measures, e.g. concentrations of metabolites and odds ratio, was added. Given the sparsity of the latter, no meta-analysis was performed.

### Pathway analysis

Subsequent to an extensive literature search and database construction (Additional file 2: Table S1), the most important metabolites retrieved, based on occurrence and significance, as so for the metabolic networks involved in childhood obesity were respectively analysed through MetaboAnalyst 5.0 (Xia et al. 2011) and MetScape 3 (Karnovsky et al. 2012) by uploading the metabolite’s KEGG identifications if such were available (Additional file 1: Table S4). Given the possibility of absence in the KEGG registry, also manual exploration was executed, i.e. for lipids and microbial metabolites. After the initiation of pathway exploration, an in-depth examination of suggested pathways was performed. Chemical classes were positioned within the pathophysiology of obesity and were compared to what has been reported on metabolic signatures associated with (paediatric) obesity and related diseases.

## Results and discussion

### Literature screening and quality assessment

**Fig. 1:**
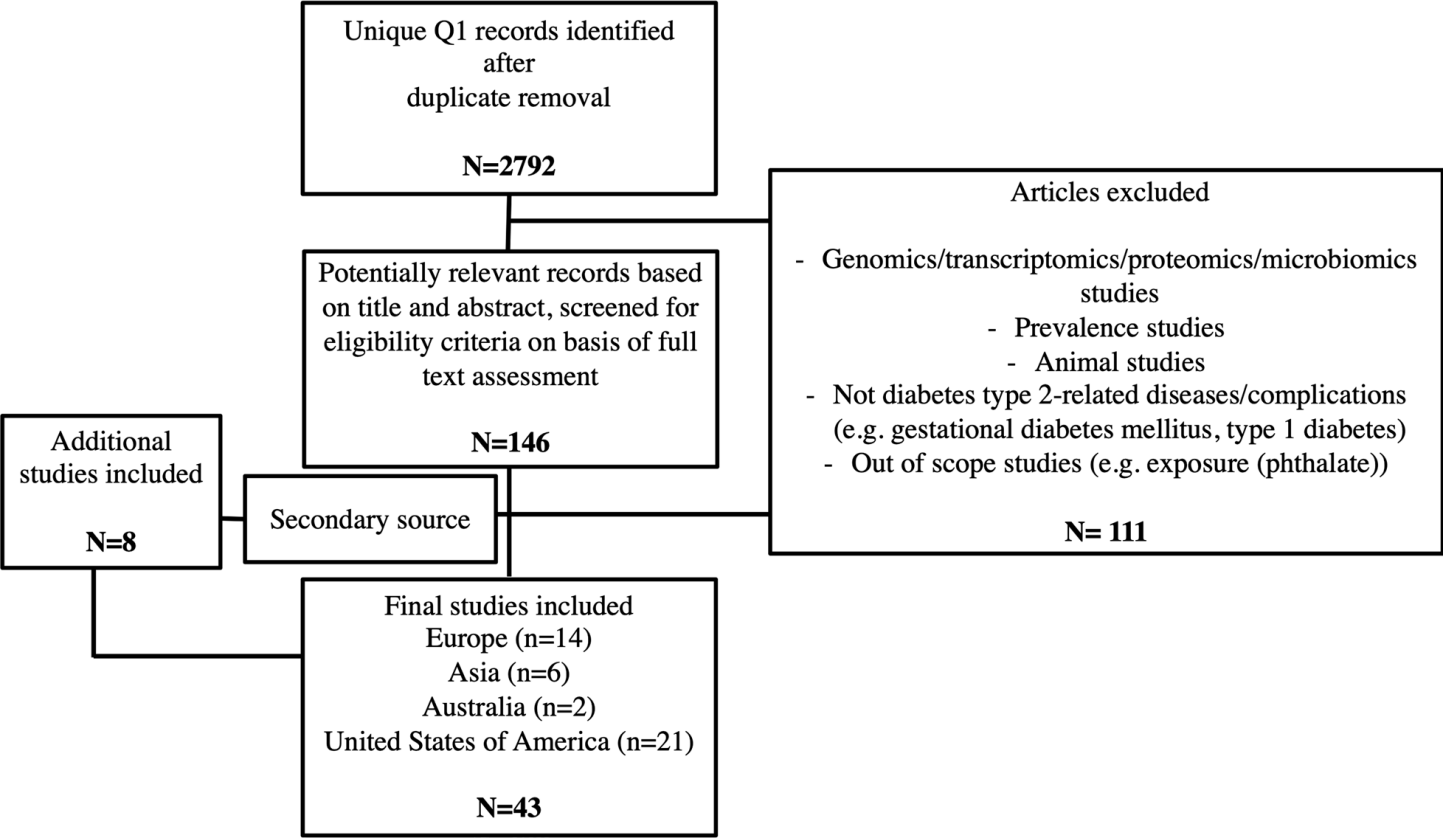
PRISMA flow diagram. PRISMA flow diagram of literature search and study selection. There were 2557, 2749 and 1875 records identified by database searching of Web of Science, Pubmed and EMBASE, respectively. After duplicate removal and Q1 filtering, 2792 articles were screened on the basis of title and abstract

The systematic literature search resulted in the identification of 43 full-text articles that met the inclusion criteria. The study selection was effectuated according to the PRISMA statement (Liberati et al. 2009) and resultant literature selection can be consulted in Fig. 1 and Additional file 1: Table S1. The quality was assessed according to the Newcastle–Ottawa Quality Assessment scale. Three studies were noted of low quality (Additional file 1: Tables S2 and S3) and therefore additionally excluded since obesity classification was not based on any local or international reference values nor BMI diagnostic criteria. Finally, 40 unique articles were included after critical selection.

### Study characteristics and data extraction

The selected studies (n = 40) and referred metabolites (n = 202) were assembled in Additional file 2: Table S1. Case–control studies (n = 22) slightly dominated over cohort studies (n = 18). The included study groups were children with (severe) obesity, (cardio)metabolically abnormal obesity (i.e. having 2 or more cardiometabolic abnormalities or meeting 1 or more criteria of cardiovascular disease risk factors), obesity with and without IR and metabolically healthy and/or normal-weight (control) subjects. In 33 studies sex-equality (48.31% ± 5.77% female %) was manifested, thereby accounting for sex differences. The number of participants ranged from 26 (Suzuki et al. 2019) to 1192 (Lau et al. 2018). Children were the study subject and ranged from 4, 5 years of age (Aristizabal et al. 2018) up to and including adolescents. If the Tanner Stage (TS) was mentioned, this was added in Table 2 and Additional file 2: Table S1. Blood predominated as matrix of choice (n = 34) with serum as most prevalent biofluid (n = 21), followed by urine (n = 3), saliva (n = 2) and adipose tissue (n = 1). Of note is that metabolomics studies on paediatric obesity using faeces, hair and nails as biological matrix were missing. By not excluding a biological matrix in particular, the blood metabolome, that already reflects a wide range of biochemical processes, was further complemented and combined with other biofluids to strive at holistic metabolome coverage and incorporate the intricate metabolic pathways at systems level. The major regional groups were America, Asia, Australia and Europe comprising 19, 6, 1 and 14 studies, respectively (Table 2 and Additional file 2). Thereby, it was possible to account for a set of plausible covariates including lifestyle (e.g. diet) and environment (e.g. air pollution) a.o. impacting the metabolism of children towards unambiguous identification of the relationship between metabolic phenotypes (metabotypes) in disease and circulating, infiltrated and excreted metabolites.

**Table 2:** Childhood obesity database of included papers

Continent	Reference paper	Study population, location and number of participants (N)	Measurement technique	Focus area	Matrix	Study design	Pubertal stage	Outcome/related disease	Diagnostics
Asia	Cho et al. (2017) Pediatric Obesity	Korea N = 184	Liquid chromatography–mass spectrometry (LC–MS)/MS and flow injection analysis (FIA)–MS/MS	Metabolomics	Urine	Cohort	> TS1	Obesity	BMI according to Korean National Growth Charts
	Kim et al. (2016) The Journal of Clinical Endocrinology & Metabolism	Korea N = 242	Gas chromatography (GC)–MS	Steroid metabolites	Serum	Case–control	> 50% TS1 and < 50% TS2, TS3 and TS4	Obesity	BMI according to Korean National Growth Charts
	Lee et al. (2018) Scientific Reports	Korea, KoCAS N = 430	LC–MS/MS	Metabolomics	Plasma	Cohort	> TS1	Obesity and diabetes type 2	BMI according to Korean National Growth Charts
	Lee et al. (2019) Scientific Reports	Korea, KoCAS-1 N = 449	FIA–MS/MS	Amino acids	Plasma	Cohort	< 5% TS1, > 45% TS2 and TS3 and > 45% TS4 and TS5	Obesity and insulin resistance	BMI according to Korean National Growth Charts
	Son et al. (2015) The Journal of Steroid Biochemistry and Molecular Biology	Korea N = 253	GC–MS	Cholesterol and other sterols	Serum	Case–control	TS1 and TS2	Obesity	BMI according to Korean National Growth Charts
	Suzuki et al. (2019) BMC Pediatrics	Japan N = 26	LC–MS	Amino acids	Plasma	Cohort		Obesity, impaired glucose tolerance and hyperuricemia	BMI according to Korean National Growth Charts
Australia	Saner et al. (2019) Metabolomics	Victoria, COBRA N = 412	^1^H-NMR	Amino acids	Serum	Cohort	> 30% TS1, 25% TS2 and TS3 and 35% TS4 and TS5	Obesity	US Centres for Disease Control (CDC) growth reference charts
Europe	Anjos et al. (2019) Journal of Proteome Research	Portugal N = 32	GC–MS and hydrophilic interaction (HI)LC–MS^2^, untargeted and 163 targets	Phospholipids	Serum	Case–control	> TS1	Obesity	Global BMI ranges (Centro Hospitalar do Baixo Vouga, Portugal)
	Hosking et al. (2019) Pediatric Diabetes	Early Bird, United Kingdom N = 150	^1^H-NMR	Amino acids	Serum	Longitudinal cohort	TS1 at baseline and ≧ TS3 at follow-up	Insulin resistance	BMI according to British 1990 standards
	Lau et al. (2018) BMC Medicine	HELIX, Multilevel European (UK, France, Spain, Norway, Greece, Lithuania) N = 1192	FIA–MS^2^ and LC–MS^2^ and ^1^H-NMR, Fingerprinting	Metabolomics	Serum and urine	Longitudinal cohort	TS1 and TS2	Not specified	WHO growth reference curves
	Mangge et al. (2016) The Journal of Nutritional Biochemistry	Austria N = 666	HPLC	Amino acids	Serum	Case–control	≧ TS3	Obesity	Austrian reference BMI percentiles and HOMA-IR
	Martos-Moreno et al. (2017) International Journal of Obesity	Spain N = 100	GC–MS^2^ and LC–MS^2^	Glycero-phospholipids	Serum	Case–control	TS1	Obesity	BMI-SDS according to Spanish standards and IOTF classification by Cole’s LMS method
	Mastrangelo et al. (2016) International Journal of Obesity	Spain N = 458	GC–MS^2^ and LC–MS^2^	Glycero-phospholipids	Serum	Case–control	TS1	Obesity	BMI-SDS according to Spanish standards
	Reinehr et al. (2015) European Journal of Nutrition	Germany N = 160	LC–MS^2^	Glycero-phospholipids	Serum	Case–control	TS1 and TS2	Obesity	BMI according to German reference data
	Rocha et al. (2018) Hormone Research in Paeditrics	Germany N = 458	Biochemical technique	Uric acid	Serum	Case–control	≧ TS1	Obesity	BMI according to German reference data
	Troisi et al. (2017) Nutrients	Italy N = 40	GC–MS	Metabolomics	Urine	Case–control	TS1, TS2, TS3 and TS4	Obesity and non-alcoholic fatty liver disease	Italian reference BMI percentiles (aged 2 to 20 years)
	Troisi et al. (2019) Nutrients	Italy N = 41	GC–MS	Metabolomics	Saliva	Case–control	≧ TS1	Obesity and non-alcoholic fatty liver disease	Italian reference BMI percentiles (aged 2 to 20 years)
	Valle et al. (2015) Pediatric Diabetes	Spain N = 86		Uric acid	Serum	Case–control	TS1	Metabolic syndrome	Spanish reference BMI percentiles (Curvas y tablas de crecimiento, 6–9 year old)
	Wahl et al. (2012) Obesity Facts	Germany N = 120	LC–MS^2^	Glycero-phospholipids	Serum	Case–control	TS1, TS2, TS3 and TS4	Obesity	IOTF classification by Cole’s LMS method
	Wijnant et al. (2020) Analytical Chemistry	Belgium N = 140	LC–MS	Metabolomics	Saliva	Case–control	≧ TS1	Obesity	BMI *z*-scores following Roelants et al. and IOTF classification by Cole’s LMS method
	Zhang et al. (2019) Journal of Adolescent Health	Finland N = 396	^1^H-NMR	Amino acids	Serum	Longitudinal cohort	TS1 and TS2 at baseline and TS5 at follow-up	Insulin resistance	Finnish reference BMI data (aged 0 to 20 years)
America	Aristizabal et al. (2017) Nutrients	Colombia N = 58	GC	FFA	Plasma	Case–control	TS1	Obesity	WC reference cut-off according to IDEFICS
	Bermudez-Cardona and Velasquez-Rodriguez (2016) Nutrients	Colombia N = 96	GC-FID	Fatty acids	Serum	Case–control	> 10% TS1, 25% TS2, TS3 and TS4 and > 60% TS5	Metabolic syndrome	WHO growth reference curves
	Butte et al. (2015) The American Journal of Clinical Nutrition	Texas N = 803	GC–MS and UPLC–MS/MS	Metabolomics	Plasma	Cohort	TS2, TS3 and TS4	Obesity	Reference BMI percentiles according to CDC growth charts for the United States of America
	Chavira-Suárez et al. (2020) PLoS ONE	Mexico N = 168	Tandem MS	Metabolomics	Serum	Case–control		Overweight and obesity	WHO growth reference curves and WHtR in *z*-scores NHANES
	McCormack et al. (2013) Pediatric Obesity	Massachusetts N = 21	Biochemical technique	Metabolomics	Serum	Case–control	TS2, TS3 and TS4	Obesity	Reference BMI percentiles according to CDC growth charts for the United States of America
	Farook et al. (2015) Pediatric Obesity	Texas N = 42	UPLC–MS/MS	Metabolomics	Serum	Case–control	TS1, TS2 and TS3	Obesity	NHANES III
	Flannagan et al. (2018) Nutrition, Metabolism and Cardiovascular disease	El Salvador, Honduras, Nicaragua, Panama, Costa Rica, Belize, the Dominican Republic and Guatemala N = 201	GC	Metabolomics	Adipose tissue	Cohort	TS1	Metabolic syndrome	BMI-*z* according to WHO Growth Reference (for children aged 5 to19 years)
	Goffredo et al. (2017) Nutrients	Connecticut N = 78	LC–MS	Branched-chain amino acids	Plasma	Case–control	TS1, TS2, TS3, TS4 and TS5	Non-alcoholic fatty liver disease	National BMI and BMI-*z* reference percentiles (Yale Pediatric NAFLD Cohort)
	Higgins et al. (2020) The Journal of Clinical Endocrinology and Metabolism	Canada N = 45	LC–MS/MS	Lipoproteins and bile acids	Serum	Cohort	< 5%% TS2, 25% TS3, 30% TS4 and > 40% TS5	Obesity	WHO growth reference curves
	Mauras et al. (2015) The Journal of Clinical Endocrinology and Metabolism	Florida N = 35	LC–MS/MS	Estrogens	Plasma	Case–control	TS1	Obesity	National reference BMI percentiles (Florida)
	Moran-Ramos et al. (2017) Scientific Reports	Mexico N = 1120	MS/MS	Amino acids	Serum	Cohort	TS1 and TS2	Obesity	Reference BMI percentiles according to CDC growth charts for the United States of America
	Newbern et al. (2014)The Journal of Clinical Endocrinology and Metabalism	North Carolina N = 82	MS^n^	Metabolomics	Plasma	Cohort	TS2, TS3, TS4 and TS5	Insulin resistance	Reference BMI percentiles according to CDC growth charts for the United States of America
	Perng et al. (2017) Paediatric Obesity	Mexico N = 238	LC–MS	Metabolomics	Serum	Cohort	35% TS1, < 10% TS2, 5% TS3 and 5% TS 4, TS5	Metabolic risk	Reference BMI percentiles (Mexico National Institute of Public Health)
	Perng et al. (2018) Pediatric Obesity	Massachusetts N = 213	Ultra-high performance (UP)LC–MS/MS	Amino acids	Plasma	Longitudinal cohort	> 65% TS1 and ≧ 30% TS2	Early adolescence	Reference BMI and BMI-*z* percentiles according to CDC growth charts for the United States of America
	Perng et al. (2019) Pediatric Research	Mexico N = 179	LC–MS	Amino acids	Serum	Longitudinal cohort	TS1 at baseline and > TS2 after 5-year follow-up	Metabolic risk	National reference BMI-*z* scores (Mexico National Institute of Public Health)
	Perng et al. (2020a; b) Obesity	Massachusetts N = 592	UPLC–MS	Metabolomics	Plasma	Case–control	> 10% TS1 and ≧ 80% TS2	Metabolic risk	Reference BMI and BMI-*z* percentiles according to CDC growth charts for the United States of America
	Short et al. (2019) The Journal of Clinical Endocrinology and Metabolism	Oklahoma N = 94	UPLC–MS	Amino acids	Plasma	Case–control	≧ TS2	Obesity	Reference BMI percentiles according to CDC growth charts for the United States of America
	Trico et al. (2017) The Journal of Clinical Endocrinology and Metabolism	Connecticut N = 78	^1^H-NMR	Amino acids	Plasma	Longitudinal cohort	> TS1	Insulin resistance	National BMI-*z* reference (the Yale Pediatric Obesity Clinic)
	Trico et al. (2019) Antioxidants and Redox Signaling	Connecticut N = 122	LC–MS/MS	Fatty acids	Plasma	Case–control	> TS1	Metabolic syndrome	National BMI-*z* reference (the Yale Pediatric Obesity Clinic)

An overview of the included studies according to the continental region of study. The first author, year of publication and name of the journal were addressed as reference. For every study, the study population, location of the study, number of participants, measurement technique, focus area of research, the matrix studied, the study design, pubertal stage of the children under study, the main outcome and method of diagnosis (in defining the groups under study) were listed

### Pathway analysis

All included compounds (n = 202), either assigned a KEGG identifier (n = 129) or, if not available, a Human Metabolome Database identifier, were listed in Additional file 1: Table S4. In the pathway analysis module of MetaboAnalyst, small *p*-values and large pathway impact generally indicate the most influenced pathways. As so, this influence results from the relative contribution of the occurrence of imported differential metabolites to the total of metabolites present in the pathway. However, for a number of lipids, including (lyso)phospholipids, glycerophospholipids and acylcarnitines, a KEGG identifier was not available. Therefore, important pathways (Additional file 1: Fig. S1), i.e. indicated by significant corrected *p*-values, high impact score or the number of hits were complemented with additional search on prevailing chemical classes. Thereby it was envisaged to take into account all plausibly relevant metabolites (Additional file 1: Table S4) and related pathways (Additional file 1: Fig. S2 and Table S5).

Recurrent metabolites were classified into overarching classes, i.e. lipids, carbohydrates and amino acids. Accordingly, every comprehensive class was further subdivided into presumably disrupted metabolic pathways and connecting those, i.e. low-grade inflammation, cell membrane fluidity, the ß-oxidation as an alternate fuel source, impaired tricarboxylic acid cycle (TCA) flux, immunometabolism and BCAAs and AAAs at the metabolic crossroads. Given few articles (n = 3) included addressed changes of hormones (including steroids and bile acids) (Mauras et al. 2015; Son et al. 2015; Kim et al. 2016) and the children studied were between 4 and 19 years of age including pre-, peri- and postpubertal stages (TS 1 up to and including 5, Table 2), hormone metabolism was not addressed.

### Lipid metabolism

**Fig. 2:**
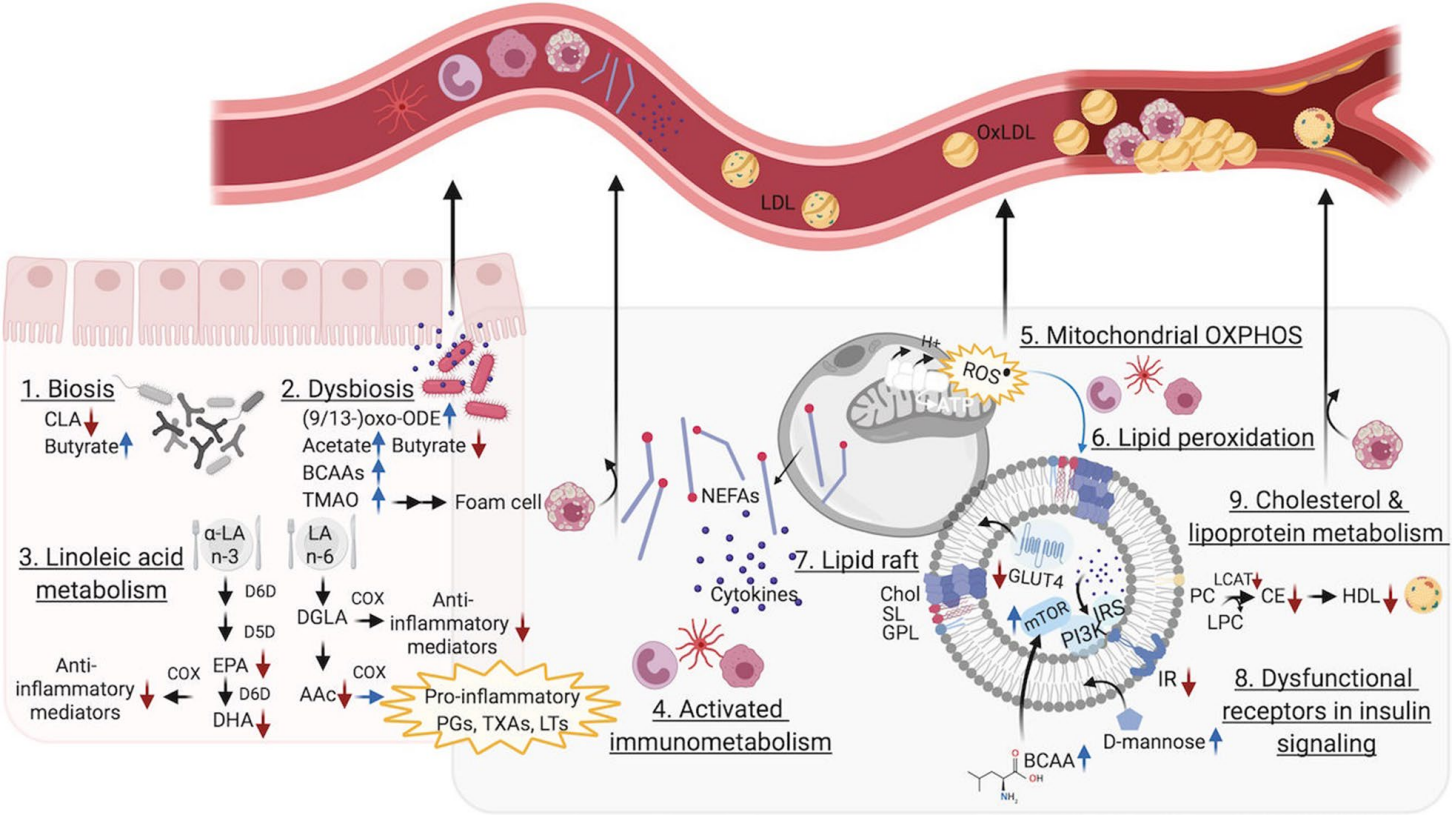
Visualisation of alterations in lipid metabolism. Overview of altered lipid metabolism and visualisation of its complex interplay resulting in a sustained oxidative environment and low-grade inflammation. Also, physiological consequences are depicted including increased intestinal permeability (linked to dysbiosis), fat mass expansion (enlarged adipocytes with rigid cell membranes) and accelerated atherosclerotic processes implied in comorbidities of obesity. Black arrows indicate reactions and movement directions, whilst red and blue arrows respectively indicate a down- and upregulation of metabolites and enzymes. Created with www.BioRender.com. CLA, conjugated linoleic acid; SCFAs, short-chain fatty acids; TMAO, trimethylamine-N-oxide; LA, linoleic acid; 9/13-oxo-ODE, 9/13-oxooctadecadienoic acid; D6D, delta-6-desaturase; D5D, delta-5-desaturase; DGLA, dihomo-gamma-linoleic acid; COX, cyclooxygenase; EPA, eicosapentanoic acid; AAc, arachidonic acid; DHA, docosahexanoic acid; PGs, prostaglandins; TXAs, thromboxane; LTs, leukotriene; NEFAs, non-esterified fatty acids; ROS, reactive oxygen species; OXPHOS, oxidative phosphorylation; PI3K, phosphatidylinositol 3-kinase; IR, insulin receptor substrate; IR, insulin receptor; Chol, cholesterol; SL, sphingolipid; GPL, glycerophospholipid; BCAA, branched-chain amino acid; PC, phosphatidylcholine; CE, cholesterol ester; HDL, high-density lipoprotein; LCAT, lecithin-cholesterol acyltransferase; LDL, low-density lipoprotein; oxLDL, oxidised low-density lipoprotein

**Fig. 3:**
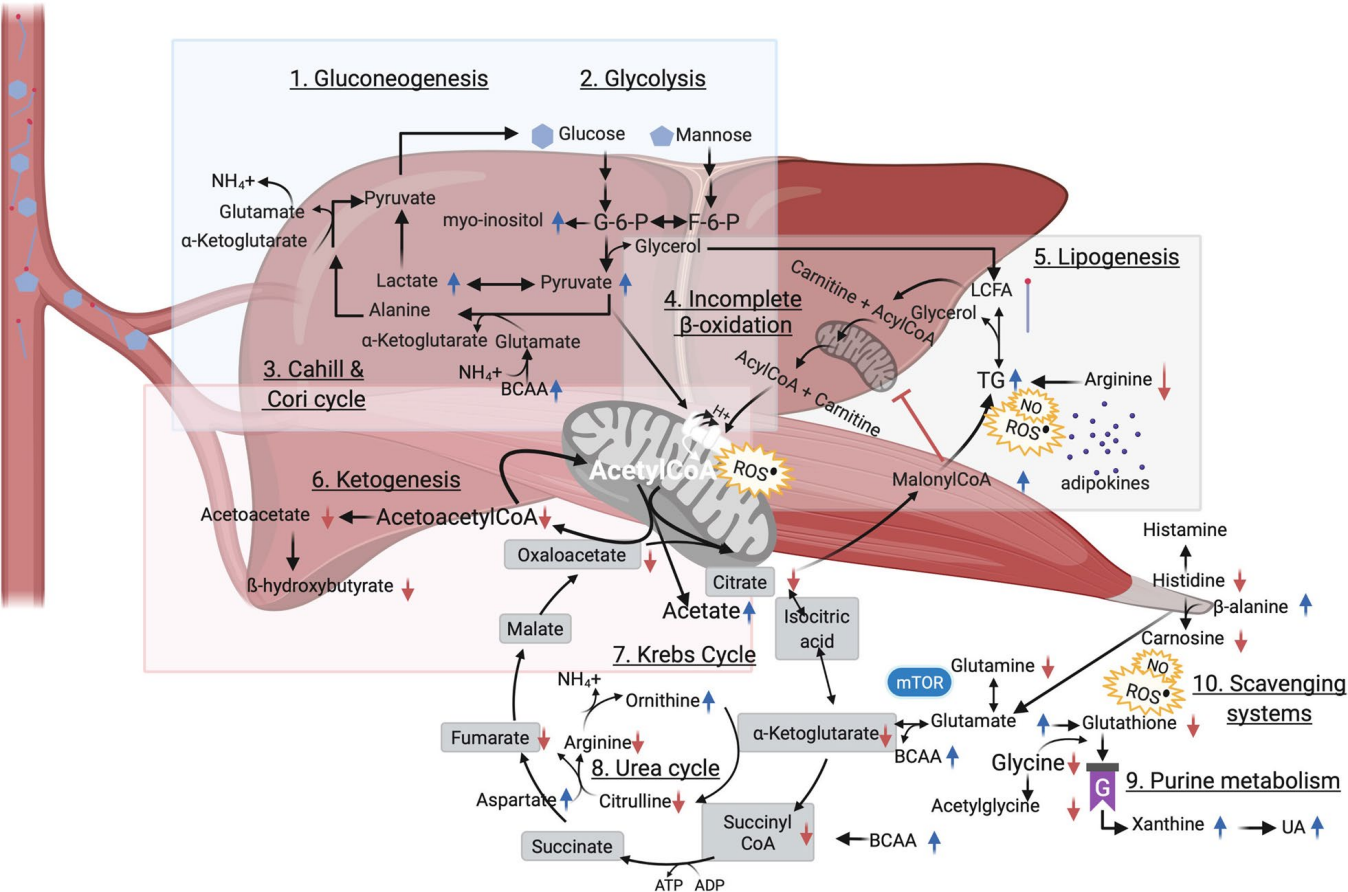
Visualisation of alterations in carbohydrate metabolism. Overview of altered carbohydrate metabolism and points of intersection with lipid and amino acid metabolism in childhood obesity. Black arrows indicate reactions, whilst red and blue arrows, respectively, indicate down- and upregulation of metabolites. Created with www.BioRender.com. G-6-P, glucose-6-phosphate; F-6-P, fructose-6-phosphate; BCAA, branched-chain amino acid; ROS reactive oxygen species; TG, triglyceride; LCFA, long-chain fatty acid; UA, uric acid; mTOR, mammalian target of rapamycin

Particularly, the fatty acids, glycero- and sphingolipids appeared the most prominent metabolite classes that showcased discrepancies and were involved in low-grade inflammation and cellular disruption (Figs. 2 and 3), which have been comprehended as fundamental biological mechanisms in the pathophysiology of obesity (Abu Bakar et al. 2015).

#### Low-grade inflammation

Aristizabal et al. (2018), Bermudez-Cardona and Velasquez-Rodriguez (2016), Butte et al. (2015), and Troisi et al. (2019) reported increased levels of palmitic acid in children with obesity but also of palmitoylglycerol and palmitoleic acid which often are incorporated into corresponding glycerolipids, i.e. triacylglycerols, boosting lipogenesis (Fig. 3, 5. Lipogenesis).

Long-chain polyunsaturated fatty acids (PUFAs) haven been associated with lower risk of diabetes and possibly prediabetes, as a new type of paediatric diabetes (Fig. 2, 3. Linoleic acid metabolism). Docosahexanoic acid (n − 3), eicosapentanoic acid (n − 3) and the eicosanoid arachidonic acid (n − 6) were observed to be lower in children with obesity (Aristizabal et al. 2018; Butte et al. 2015; Flannagan et al. 2018), which could relate to anti-inflammatory properties ascribed to the n − 3 series and to competition of cyclooxygenase enzyme activity due to the high stress environment that is characteristic of obesity. Moreover, the enzymatic activity of delta-5-desaturase (D5D), responsible for the conversion of a.o. dihomo-gamma linoleic acid into arachidonic acid (Fig. 2, 3. Linoleic acid metabolism), has previously been observed reduced in diverse metabolic disorders, e.g. non-alcoholic fatty liver disease, and has been associated with underlying IR and oxidative stress (Tosi et al. 2014). Dihomo-gamma-linoleic acid and arachidonic acid produce different series of thromboxane, i.e. respectively anti-inflammatory and pro-inflammatory. The increased levels of dihomo-gamma-linoleic acid observed in four studies (Aristizabal et al. 2018; Bermudez-Cardona and Velasquez-Rodriguez 2016; Butte et al. 2015; Flannagan et al. 2018), paralleling reduced conversion into anti-inflammatory products and the inverse for arachidonic acid, suited the acknowledged oxidative stress environment in childhood obesity (Tosi et al. 2014). In line with the upregulated enzyme activity, linoleic acid (n − 6) was decreased in five studies on blood (Anjos et al. 2019; Perng et al. 2020a; Aristizabal et al. 2019; Bertoli et al. 2015; Trico et al. 2017) (Fig. 2, 3. Linoleic acid metabolism). Yet, contrasting observations were mentioned by Trico et al. (2019) who reported on increased levels of linoleic acid and its oxidised derivates 9- and 13-oxo-ODE in the plasma of children with obesity, reflecting advanced oxidative state in parallel to lipotoxicity in the development of insulin resistance (Zhao et al. 2016). These oxidised metabolites have been associated with lower linoleic acid-conjugating gut bacteria, which was referred to as a detoxification mechanism to reduce systemic low-grade inflammation (Trico et al. 2019) (Fig. 2, 1. Biosis).

An increment of lysophospholipids, e.g. lysophosphocholine which is characterised with pro-inflammatory effects, was observed by Mastrangelo et al. (2016) and related to their negative interaction with G protein-coupled receptors in inflammation processes and insulin production and sensitivity (Bas et al. 2016). This parallels a reduction in the inhibiting effect of PUFAs like docosahexanoic acid on the hydrolytic action of phospholipase A2 in individuals suffering from overweight (Lau et al. 2018; Butte et al. 2015; Anjos et al. 2019; Farook et al. 2015; Cho et al. 2017; Lee et al. 2018; Reinehr et al. 2015), which also is in consistency with adult findings (Bas et al. 2016). Alternatively, changes in the enzyme activity of lecithin-cholesterol acyltransferase, an enzyme that converts free cholesterol to its ester substitutes (Fig. 2, 9. Cholesterol & lipoprotein metabolism), and promotes the formation of high-density lipoprotein (Ng 2012; Rousset et al. 2009) could be implicated.

#### Cell membrane fluidity

Oxidative processes go hand in hand with alterations in dynamic cell structures and functions of insulin-sensitive tissues (Perona 2017), e.g. cellular membrane fluidity of pancreatic cells and adipocytes. For example lipid peroxidation renders cellular membranes more rigid (Fig. 2, 6. Lipid peroxidation), whereas PUFAs could be addressed in restoring membrane responsiveness as shown in skeletal muscle membranes (Perona 2017). When cell membranes are characterised by a high abundance of cholesterol, sphingolipids and glycerophospholipids (all bearing predominantly saturated fatty acids), these are referred to as microdomains or lipid rafts and contribute to tight packaging (Perona 2017) (Fig. 2, 7. Lipid raft). Cho et al. (2017), Lau et al. (2018) and Lee et al. (2018) reported on alterations in sphingolipid metabolism in children with obesity (Additional file 1: Fig. S1), i.e. both increments and reductions of sphingomyelins were observed. Sphingomyelins are ubiquitous membrane constituents and have been ascribed previously as independent predictors of IR (Perona 2017). The observed reductions mainly imparted saturated sphingomyelins, e.g. sphingomyelin 16:0 (Lau et al. 2018; Cho et al. 2017; Lee et al. 2018), and could result of more incorporation of the latter into microdomains. Increased levels however especially concerned the unsaturated share, e.g. sphingomyelin 16:1 and 18:1 (Lau et al. 2018). In this context, also elevations of palmitoleic and oleic acid were reported by Bermudez-Cardona and Velasquez-Rodriguez (2016) and Troisi et al. (2017) that could be related to ceramide synthesis and its association with membrane rigidity (Zheng et al. 2006). In particular, C16:0 ceramide, matching the increased presence of palmitic acid (Aristizabal et al. 2018; Bermudez-Cardona and Velasquez-Rodriguez 2016; Troisi et al. 2019; Anjos et al. 2019) and decreased sphingomyelin 16:0, have been observed as negative regulators of insulin signaling and inhibitor of mitochondrial fatty acid ß-oxidation and as such, identified as important mediators of obesity-derived IR and impaired ß-oxidation (Fucho et al. 2016).

Anjos et al. (2019) noted a decrease of all measured phosphatidylinositol species in the serum of children with obesity. Among the glycerophospholipids in microdomains, phosphatidylinositol constitutes the major building block in lipid rafts that has been demonstrated in target tissues of IR (Boini et al. 2017). Besides, phosphatidylinositol anchors a plurality of membrane receptors. The corollary to this and alterations in physicochemical properties of the cell membrane structure is that the functional integrity of receptors and thus signaling pathways could be influenced (Perona 2017). In the case of obesity, the impairment in uptake of glucose and insulin action could be a reflection of such functional dependence of both glucose transporters (GLUT) and the insulin receptor, respectively (Fig. 2, 8. Dysfunctional receptors in insulin signaling). Taken together, several metabolites related to lipid metabolism are in favor of the cell membrane hypothesis of IR and may infer, even at early age, the existence of a vicious continuum.

#### ß-oxidation as an alternate dysfunctional fuel source

When hyperglycemia is present, the amount of non-esterified fatty acids in plasma should be reduced following insulin-mediated suppression of both endogenous gluconeogenesis, lipolysis and ß-oxidation. Despite, all included studies that reported on non-esterified fatty acids noted increased circulating values. This could result from their release by saturated adipocytes into the blood stream, leading to lipotoxicity, and in turn, interfering in a vicious way with insulin-responsive metabolic pathways (Chavira-Suárez et al. 2020). This is, higher levels of non-esterified fatty acids have been reported to activate immune and oxidative stress pathways (Fig. 2, 4. Activated immunometabolism) and as such, could affect insulin signalling and glucose transport triggering IR in skeletal muscle and liver (Fucho et al. 2016; Boini et al. 2017). Furthermore, when fats are addressed as an alternative energy source, degradation of triglycerides to glycerol and FFA needs to occur before ß-oxidation can take place. However, lowered glycerol concentrations were observed in children with obesity by Troisi et al. (2019), which could point towards decreased lipolysis and subsequent ß-oxidation, and/or increased glycerol utilisation during upregulated lipogenesis (Fig. 3, 4. Incomplete ß-oxidation and 5. Lipogenesis).

Complete ß-oxidation renders acetyl-coenzyme A (CoA) that is further used during the biosynthesis of acylcarnitines, the so-called regulators of ß-oxidation (Martos-Moreno et al. 2017). Of note is that the serum acylcarnitine profile is reflected by its reservoir in skeletal muscle. Given skeletal muscle IR is the primary feature in obesity, acylcarnitines are considered relevant biomarkers of early occurring obesity-related IR. In congruency with observations made in adult reports (Rangel-Huerta et al. 2019), diverse acylcarnitines, e.g. malonyl-, propionyl-, valeryl-, octanoyl-, nonaoyl- oleoyl- and palmitoylcarnitine, were found consistently higher in studies on children with obesity (Lau et al. 2018; Butte et al. 2015; Farook et al. 2015; Cho et al. 2017; Lee et al. 2018; Martos-Moreno et al. 2017; Perng et al. 2018, 2020b; Moran-Ramos et al. 2017) (Fig. 3, 4. Incomplete ß-oxidation).

### Carbohydrate metabolism

A variety of metabolites that belong to the central carbon metabolism, i.e. glycolysis, the Krebs cycle and acylcarnitine metabolism, showed prominent alterations in children with obesity.

#### Impaired Krebs cycle flux

Next to ß-oxidation, glucose oxidation or “glycolysis” is a physiologically important process for yielding energy. There exists a reciprocal regulation, which is known as “the glucose-fatty acid cycle”, to maintain adequate acetyl-CoA levels and Krebs cycle activity, reflecting their interrelatedness. Acetyl-CoA can result from complete ß-oxidation, which is impaired in obesity, as from ketone body catabolism, carbohydrate and amino acid metabolism (Fig. 3). The perturbations of ß-oxidation are presumed to be counteracted by switching to carbohydrate substrates. In congruency, the excess energy intake characteristic of obesity was reflected by elevations in simple sugars (mono- and disaccharides) in blood (Fig. 3, 2. Glycolysis) as well as saliva and urine of children with obesity in diverse studies (Butte et al. 2015; Troisi et al. 2017, 2019; Hosking et al. 2019) and was indicative of pronounced stimuli towards a glycolytic metabolism. In addition, some simple sugars, such as d-mannose, serve as essential building blocks of glycoproteins like the insulin receptor (Lee et al. 2016). Elevations in d-mannose could therefore be related to a declined incorporation into and/or downregulation of insulin receptors (Fig. 2, 8. Dysfunctional receptors in insulin signaling) and has been suggested as contributor to the development of IR and diabetes type 2 in adults (Lee et al. 2016).

TCA cycle intermediates were altered in childhood obesity, i.e. decreased blood values of citric acid and α-ketobutyrate were observed by Butte et al. (2015), Hosking et al. (2019) and Troisi et al. (2017). Citrate might be escaping the mitochondria to enable its conversion into malonyl-CoA (Fig. 3, 4. Incomplete ß-oxidation and 5. Lipogenesis). This could be reflective of increased rates of lipogenesis and concordant overproduction of ROS, pro-inflammatory prostaglandins and adipokines and altered nitric oxide (Chavira-Suárez et al. 2020), thereby inflecting immune response (Fig. 3, 5. Lipogenesis). Studies by Hosking et al. (2019), Martos-Moreno et al. (2017), Mastrangelo et al. (2016) and Saner et al. (2019) showcased reduced ketone body production, i.e. ß-hydroxybutyrate and acetoacetate (Fig. 3, 6. Ketogenesis), supporting impaired TCA cycle flux. It is worth mentioning that insulin is known to exert antiketotic effects. Therefore, reduced ketone body anabolism (Mastrangelo et al. 2016; Martos-Moreno et al. 2017; Hosking et al. 2019; Saner et al. 2019) might be an early indicator of hyperinsulinemia as so for metabolic health going forward since reduced capacity to generate ketones might have significant long-term implications for body weight regulation.

Increased measures of pyruvate but also of lactate were repeatedly reported in children with obesity (Mastrangelo et al. 2016; Martos-Moreno et al. 2017; Hosking et al. 2019). Pyruvate is a key intermediate of glycolysis and serves as precursor for gluconeogenesis and the biosynthesis of glycerol, fatty acids and non-essential amino acids (Fig. 3, 1. Gluconeogenesis, 2. Glycolysis and 5. Lipogenesis). Elevated levels of pyruvate suggest a deficiency in the pyruvate dehydrogenase enzyme (complex), which is necessary in producing acetyl-CoA going forward. Increments of lactate were also noted (Fig. 3, 2. Glycolysis), which may reflect dysregulations in central carbon metabolism and tend to direct metabolism towards fermenting conditions in children with obesity, termed the “aerobic glycolysis” or “Warburg effect” (Wu et al. 2016). Accordingly, from a physiological point of view, obesity goes hand in hand with adipocyte hypertrophy which is associated with local hypoxia boosting lactate production (Longo et al. 2019).

The breakdown of glucogenic amino acids can deliver acetyl-CoA as a compensatory increase of glycoylis, whereby further providing linkage of carbohydrate and amino acid metabolism. In particular, alanine may act through the “glucose-alanine” or Cahill cycle as a contributor to the observed increase of pyruvate and lactate levels (Suzuki et al. 2019; Mastrangelo et al. 2016; Martos-Moreno et al. 2017; Moran-Ramos et al. 2017; Hosking et al. 2019; Zhang et al. 2019; Short et al. 2019; Perng et al. 2019), supported by increased levels of urea (Troisi et al. 2019). Increased flux through the Cahill and Cori cycles in liver and skeletal muscle parallels inefficient inhibition of hepatic gluconeogenesis (Fig. 3, 1. Gluconeogenesis and 3. Cahill & Cori cycle) as well as enhanced pyruvate oxidation secondary to reduced ß-oxidation and impaired glucose utilisation. As such, high lactate, pyruvate and alanine may form a characteristic signature in children with obesity and IR.

#### Energy demanding processes in immunometabolism

Both Perng et al. (2017) and Troisi et al. (2019) detected higher myo-inositol, the most prominent form of inositol’s stereoisomers. Myo-inositol serves as a precursor molecule to membrane-associated phosphatidylinositols (Additional file 1: Fig. S2, phosphatidylinositol phosphate metabolism) and secondary (immunological) messengers like inositol triphosphate. Besides, the downstream metabolisation processes, e.g. epimerisation of myo-inositol, are insulin-dependent, hinting towards the early existence of IR. Alternatively, since myo-inositol can be formed from glucose (Fig. 3, 2. Glycolysis), enhanced immunometabolism in childhood obesity and thus increased demand of myo-inositol could also be serving as a stimulant to upstream gluconeogenesis as well as boosting glycolytic machinery during inflammation.

Obesity has been acknowledged with dysbiosis impacting low-grade inflammation, energy metabolism and expanding fat mass. Higher levels of short-chain fatty acids have been reported before in faeces (Schwiertz et al. 2010) and were noted as well for acetate in urine by Lau et al. (2018), which is in line with a more anaerobic environment including microbial colonic fermentation processes in obesity (Fig. 2, 2. Dysbiosis). Despite, Hosking et al. (2019) and Saner et al. (2019) observed significantly negative associations of serum acetate with IR, for which a premise could be sought in the peripheral metabolisation of acetate. This is, either a consequence of impaired Krebs cycle flux (Fig. 3, 7. Krebs cycle) and/or an altered bifidobacterial production and metabolisation of acetate and thus, inferring suppression of butyrogenic members of the gut microbiota by reducing acetate consumption in children with obesity (Fig. 2, 2. Dysbiosis) and/or favoring the role of acetate as a substrate for cholesterol synthesis. Furthermore, urinary correlations of acetate with succinate and formate have been reported (Lau et al. 2018), thereby underpinning the impaired Krebs cycle flux hypothesis and providing linkage with bacterial metabolism (Rombouts et al. 2021).

Moreover, the microbial metabolite trimethylamine-*N*-oxide has been previously seen augmented in diabetes type 2 patients and referred to as proatherogenic substance due to its modulation of cholesterol metabolism and subsequent contribution to increased formation of foam cells (Fig. 2, 2. Dysbiosis). Hosking et al. (2019) and Lau et al. (2018) reported discrepant findings, i.e. decreased serum and increased urinary levels, respectively, which could be resulting from biological variations of dietary compositions. This metabolite could promote reduction in CYP7A1 activity, which is a vital enzyme in the synthesis of bile acids, forms a rate limiting step in the catabolism of cholesterol and of which its variant with reduced activity has been ascribed to enhance atherosclerosis (Canyelles et al. 2018).

It is thus reasonable that the composition of gut microbiota during early life influences the development of overweight and obesity in children and further progression into related diseases (Gilbert et al. 2016). Indeed, its composition and function are shaped by host genetics, early life environment and dietary reciprocal interactions with significant involvement of metabolome compositions and thus conceivable contribution of the latter in disease susceptibility (Snijders et al. 2016).

### Amino acid metabolism

Besides carbohydrates, also amino acids are of necessitate importance to immune cells. An overall upsurge of amino acids was noted and their potential role in obesity and its sequelae were elaborated in the following section. In particular, the increase of BCAAs were inferred to differentiate prepubertal children with obesity and IR from those without IR.

#### Amino acids and immune function

Histidine metabolism (Additional file 1: Fig. S1) is ascribed an essential role in immunometabolism as decreased values hint towards an activated immune response. Histidine, the precursor of the biogenic histamine, was globally found to be lowered in a consequent manner starting at young age by Cho et al. (2017), Saner et al. (2019), Short et al. (2019) and Zhang et al. (2019) in Asia, Australia, America and Europa, respectively. A decrease in carnosine was observed by Cho et al. (2017) and ß-alanine was observed higher in children with obesity by Short et al. (2019) (Fig. 3, 10. Scavenging systems). In line with decreased values of histidine, elevated levels of the gut microbially produced histidine metabolite, i.e. imidazole propionate, were found in adults with diabetes type 2 and involved directly in IGT and insulin signaling.

Arginine is associated through the urea cycle with the occurrence of ornithine and citrulline. Overall, increased aspartate and ornithine and decreased arginine and citrulline values (Fig. 3, 8. Urea cycle) were observed (Suzuki et al. 2019; Butte et al. 2015; Cho et al. 2017; Lee et al. 2018; Chavira-Suárez et al. 2020; Martos-Moreno et al. 2017; Moran-Ramos et al. 2017; Short et al. 2019; Perng et al. 2019; Tepper et al. 2016; Mangge et al. 2016). A disrupted metabolism of arginine and proline (Additional file 1: Fig. S1) has been associated with increased oxidative stress and generation of triglycerides (Lay et al. 2014). Arginine has been ascribed positive modulating functions in the expression of enzymes involved in ß-oxidation and would stimulate lipolysis as well as inhibit gluconeogenesis (McKnight et al. 2010), whilst obesity is characterised by opposite directions of those pathways. In this regard, a reduced level of arginine, which forms a substrate for nitric oxide synthase, has been related to increased production of ROS and nitric oxide, especially in the mitochondria of the liver (Fig. 3, 5. Lipogenesis). The catabolism of arginine thus either results in ornithine and urea by arginase activity or in nitric oxide (Zhao et al. 2020) which both stimulate immune response.

Several authors observed increased glutamate (Suzuki et al. 2019; Lau et al. 2018; Butte et al. 2015; Lee et al. 2018; Short et al. 2019) and decreased glutamine levels (Cho et al. 2017; Reinehr et al. 2015; Zhang et al. 2019; Short et al. 2019) in children with obesity but also in children suffering from metabolic syndrome and non-alcoholic fatty liver disease (Short et al. 2019; Goffredo et al. 2017). Conventionally, glutamine is used by aerobic proliferating cells for biomass production through the TCA cycle. Consequently, a diverted metabolism in hypoxic cells as seen by an upregulated production of lactate from glucose might parallel rewiring of glutamine metabolism. This could then further contribute to increased lipogenesis, i.e. reductive carboxylation of α-ketoglutarate (Fig. 3, 10. Scavenging systems). Depletion of hepatic enzymes that catalyse the conversion of glutamate into the scavenger molecule glutathione has been acknowledged in paediatric obesity (Pastore 2012). In line herewith, a decreasing trend of glycine and its acetylated form was observed (Suzuki et al. 2019; Butte et al. 2015; Cho et al. 2017; Zhang et al. 2019; Short et al. 2019).

In the context of immunometabolism, purine and pyrimidine metabolism (Additional file 1: Fig. S1) are worth mentioning as both glycine and glutamine are consumed during biosynthesis of purines like guanine (Fig. 3, 9. Purine metabolism), substantiating observed lower values of the latter two (Butte et al. 2015; Cho et al. 2017; Reinehr et al. 2015; Zhang et al. 2019; Short et al. 2019). In conjunction herewith, Butte et al. (2015), Perng et al. (2017), Rocha et al. (2016), Suzuki et al. (2019) and Valle et al. (2015) observed hyperuricemia to be associated with alterations for a diverse array of metabolic features. For instance, uric acid contributes to an oxidative stress environment promoting endothelial dysfunction and inflammation. In specific, serum uric acid has significantly been correlated to C-reactive protein and interleukin-6 levels (Perng et al. 2017; Valle et al. 2015). Uric acid is even referred to as alarmin, indicative for tissue damage including liver, which may hint towards its involvement in systemic low-grade inflammation and non-alcoholic fatty liver disease. Remarkably, as Suzuki et al. (2019) and Valle et al. (2015) studied 6 to 9-year and 9 to 10-year old children, a deranged profile of amino acids and hyperuricemia might entail the development of risk factors that accompany metabolic syndrome and cardiovascular disease, even before pubertal onset. Furthermore, Butte et al. (2015) noted increased values of the purine xanthine, whilst Perng et al. (2017) observed decreased levels of the pyrimidine thymine, which respectively serve as direct and indirect precursor molecules of the final metabolite of purine metabolism, i.e. uric acid, thereby pointing towards an accelerated purine catabolism in childhood obesity.

#### Branched-chain and aromatic amino acids at the metabolic crossroads

Decreased muscle utilisation, i.e. BCAA catabolism, could explain characteristic plasma elevations of BCAAs as they may compete with glutamate for uptake into tissue via the neutral amino acid transporters. Of note is that these increments have been widely observed in pediatric obesity, especially when IR was present (Lau et al. 2018; Butte et al. 2015; Troisi et al. 2019; Trico et al. 2017; Lee et al. 2018; Martos-Moreno et al. 2017; Perng et al. 2017, 2018, 2019; Moran-Ramos et al. 2017; Hosking et al. 2019; Zhang et al. 2019; Rupérez et al. 2020). Even more, the positive association between elevated BCAAs and IR was reported as consistent with adult studies before by Zhao et al. (2016), underpinning their alarming rise in early life.

The BCAAs leucine and isoleucine have been related to the PI3K–AKT–mTOR pivotal nutrient-sensitive signaling pathway that is implied in ß-cell growth and proliferation as well as its downstream effector functions including glucose uptake by GLUT4 in insulin sensitive tissues, i.e. liver, muscle and adipose tissue (Mastrangelo et al. 2016; Mangge et al. 2016) (Fig. 2, 8. Dysfunctional receptors in insulin signaling).

Kynurenine and kynurenic acid, resulting from one of the three major biochemical conversions tryptophan undergoes (Additional file 1: Fig. S2, tryptophan metabolism), were reported consistently higher in children with obesity according to Butte et al. (2015) and Perng et al. (2020b). Those metabolites have been associated to immune cell activation and low-grade systemic inflammation. Besides, a decrease in indole-3-propionic acid was noted by Farook et al. (2015), inferring reduced gut microbial transformation of tryptophan. As the latter metabolite was ascribed free-radical scavenging and oxidative stress reducing properties (Abildgaard et al. 2017), its decrease further underpins the presumed association with oxidative immunometabolism and relates to the observed dysbiosis in obesity (Pedersen et al. 2016).

The AAA tyrosine was reported as biomarker for future insulin resistance and metabolic risk before by Zhao et al. (2016). In this regard, elevated levels of phenylalanine and its hydroxylation product, tyrosine, provided strong relevance as predisease metabolites predictive of the development of cardiovascular disease and diabetes type 2 (Suzuki et al. 2019; Mangge et al. 2016) susceptibility from early age on (Suzuki et al. 2019; Lee et al. 2018; Martos-Moreno et al. 2017; Perng et al. 2018, 2019; Short et al. 2019; Mangge et al. 2016). As in the case of glutamate, AAAs could also compete for neutral amino acid transporters with BCAAs, leading to the accumulation of both. Notably, the consistent tendency of AAAs and BCAAs and, in particular their combination rendered the best performing principal component in the study of Butte et al. (2015) regarding the classification of obesity with IR. Additionally, increased values of methionine, cystathionine and cysteine in blood were observed (Reinehr et al. 2015; Moran-Ramos et al. 2017; Frohnert and Rewers 2016; Suzuki et al. 2019), but not by Troisi et al. (2017) and Cho et al. (2017) in urine. The characteristic oxidative environment in obesity is assumed to trigger catabolism of methionine, namely cysteine and methionine metabolism (Additional file 1: Fig. S1), yet not up to the point of glutathione production given the decreased levels of glycine. In parallel, the generation of cysteine/cystine was increased, which could act as an inhibitor for tyrosine aminotransferase and thus lead to the accumulation of tyrosine and its precursor phenylalanine. The rationale for this event to occur in the obese state is further underpinned by the postulation that inhibition of tyrosine aminotransferase goes together with the attenuation of α-ketoacid dehydrogenase enzyme activities, corresponding with its reduced activity in a variety of insulin-sensitive tissues (Adams 2011).

BCAAs could serve as substrates for gastrointestinal microbial fermentation into products like short-chain fatty acids which protect intestinal barrier integrity (Quan et al. 2020), inferring an obesity-induced dysbiotic drift and increased gastrointestinal membrane permeability (Fig. 2, 2. Dysbiosis). In line herewith, short-chain fatty acids have been appointed a role in microbial production of myristoleic acid, an unsaturated long-chain fatty acid that has been attributed anti-obesity effects through brown tissue activation (Quan et al. 2020). Thus, a bidirectional interaction of an obesogenic environment and the microbiome is very likely to occur already in childhood, i.e. metabolic interactions shaping the host’s microbiome and gut microbes modulating host metabolism. Furthermore, increments of BCAAs in insulin-resistant individuals with obesity have been correlated to a specific gut microbiome (Lee et al. 2019). This is, an enriched biosynthetic potential for BCAAs on the one hand and, on the other hand, the deprivation of genes that encode bacterial inward transporters for BCAAs to systemic circulation by an increased gut permeability (Pedersen et al. 2016).

## Conclusion

Metabolomics studies on childhood obesity have so far enabled to shed light on the existence of differentiating metabolic profiles. Both systemic low-grade inflammation and hyperglycemia, which instigate disease development, were reflected in aberrations of several metabolites related to lipid, carbohydrate and amino acid. Remarkably, the main metabolites at the crossroad of dysregulated metabolic pathways could be tracked down to one central disturbance, i.e. impending IR. However, as internationally agreed reference measures for (ab)normal insulin sensitivity during childhood are still lacking, this systematic review assembled metabolite patterns of obesity and -related IR towards prompt signalisation of high-risk phenotypes.

In essence, a pronounced higher, yet inefficient utilisation of carbohydrates and fatty acids was evinced which may be attributed to the cell membrane hypothesis of IR and mitochondrial toxicity. In congruency, impaired TCA cycle flux and ß-oxidation rise already from early on. In general, the central carbon metabolism was shifted towards hypoxic conditions in children with obesity as high pyruvate, lactate and alanine served as markers of disrupted metabolism occurring early in life. Moreover, the ubiquitous elevation of BCAAs in obesity was involved in dysregulated ß-oxidation, stimulated gluconeogenesis, reduced ketogenesis and increased gastrointestinal membrane permeability and could therefore be a characteristic differentiating feature for children with obesity and resistance to insulin versus the non-resistant ones.

Among the heterogeneities observed between the studies reviewed, the most important influencing factors with regard to the metabolome and lipidome were considered ethnicity, diet and physical activity. By globally assessing the childhood obesity epidemic, i.e. the inclusion of studies performed in America, Asia, Australia and Europe, it was attempted to account for such covariates impacting the metabolism of children. Also, a high number of studies that were included in this systematic review had a rather small sample size which might burden the false discovery rate of the appointed metabolites as potential candidate biomarkers in childhood obesity and its associated comorbidities. However, as most metabolites were retrieved in multiple included papers, their reproducibility was considered acceptable. Thus, despite such limitations, several metabolites and in particular those belonging to lipid and amino acid metabolism could be ascribed a role in future risk of metabolic disease onset given their recurrence in diverse metabolic pathways. Therefore, prolonged longitudinal research, which is to date very scarce, is warranted to confirm their value as predictive biomarkers in early risk-stratification as well as target intervention studies to validate rewiring of dysfunctional pathways, reverting metabolic disease onset and thus confirming their potential in tailored interventions.

## Supplementary Information

**Additional file 1.** **Table S1.** Pragmatic database search strategy according to the PICO framework. **Table S2.** Quality assessment of the included case-control (n=22) and cohort studies (n=20) using the Newcastle-Ottawa Scale. **Table S3.** Quality assessment of the included case series (n=1) using an adjusted Newcastle-Ottawa Scale. **Table S4.** Compound database for MetScape 3. **Table S5.** Pathway analysis using MetaboAnalyst 5.0. **Figure S1.** Visualisation of pathway analysis, using MetaboAnalyst 4.0. **Figure S2.** The metabolic network and pathway mapping.

**Additional file 2.** **Table S1.** Database including all data extracted from selected studies. Database concerning altered metabolites in paediatric patients with overweight and obesity in separate excel file, including first author and title, year of publication, continent, country of the study, study design, sample size, diagnostic criteria, characteristics of the study populations (age, Tanner stage and sex), analytical technique, biological matrix studied and quantitative findings, if these were available.

## Data Availability

The dataset(s) supporting the conclusions of this article are available in the Additional Information section and constructed metabolite database provided as additional Excel file (Additional file 2).

## Abbreviations

IFG: Impaired fasting glucose
IGT: Impaired glucose tolerance
IR: Insulin resistance
HOMA-IR: Homeostatic model assessment of insulin resistance
QUICKI: Quantitative insulin sensitivity index
OGTT: Oral glucose tolerance test
BMI: Body mass index
BCAA: Branched-chain amino acid
AAA: Aromatic amino acid
PICO: Patient–intervention–comparison–outcome
PRISMA: Preferred reporting items for systematic reviews and meta-analyses
^1^H-NMR: Proton nuclear magnetic resonance
ChEBI: Chemical entities of biological interest
KEGG: Kyoto Encyclopedia of Genes and Genomes
TS: Tanner stage
PUFA: Polyunsaturated fatty acid
D5D: Delta-5-desaturase
ROS: Reactive oxygen species
TCA: Tricarboxylic acid
GLUT: Glucose transporter
PI3K: Phosphatidylinositol-3-kinase
ATP: Adenosine triphosphate
CoA: Coenzyme A
mTOR: Mammalian target of rapamycin
