# Supplementary material for: Paediatric obesity: a systematic review and pathway mapping of metabolic alterations underlying early disease processes
Source: Mol Med. 2021 Nov 6;27:145. doi: 10.1186/s10020-021-00394-0 (PMC8571978; doi:10.1186/s10020-021-00394-0)
Supplement: Supplementary file 1 — Additional file 1. Table S1. Pragmatic database search strategy according to the PICO framework. Table S2. Quality assessment of the included case-control (n=22) and cohort studies (n=20) using the Newcastle-Ottawa Scale. Table S3. Quality assessment of the included case series (n=1) using an adjusted Newcastle-Ottawa Scale. Table S4. Compound database for MetScape 3. Table S5. Pathway analysis using MetaboAnalyst 5.0. Figure S1. Visualisation of pathway analysis, using MetaboAnalyst 4.0. Figure S2. The metabolic network and pathway mapping. [file 10020_2021_394_MOESM1_ESM.pdf]

Paediatric obesity: a systematic review and pathway mapping of metabolic alterations underlying early disease processes

Margot DE SPIEGELEER[a], Ellen DE PAEPE[a], Lieven VAN MEULEBROEK[a], Inge GIES[b] , Jean DE SCHEPPER[b,c] and Lynn VANHAECKE[a,d,*]

[a] Laboratory of Chemical Analysis, Department of Translational Physiology, Infectiology and Public Health, Faculty of Veterinary Medicine, Ghent University, Salisburylaan 133, 9820 Merelbeke, Belgium;

[b] KidZ Health Castle, Universitair Ziekenhuis Brussel, Vrije Universiteit Brussel, Laarbeeklaan 101, 1090 Brussel, Belgium;

[c] Department of Internal Medicine and Pediatrics, Faculty of Medicine and Health Sciences, Ghent University, Corneel Heymanslaan 10, 9000 Ghent, Belgium;

[d] Institute for Global Food Security, School of Biological Sciences, Queen's University, University Road, Belfast, BT7 1NN, United Kingdom.

[*]Corresponding Author

Prof. dr. Lynn Vanhaecke

Ghent University – Faculty of Veterinary Medicine

Department of Translational Physiology, Infectiology and Public Health - Laboratory of Chemical Analysis

Salisburylaan 133, B-9820 Merelbeke, Belgium

Tel: +32 9 264 74 57; Fax: +32 9 264 74 92

E-mail: Lynn.Vanhaecke@UGent.be

Table of content

Additional Tables and Figure Titles (in bold) and Legends

Additional File 1:

Table S1. Pragmatic database search strategy according to the PICO framework

Table S2. Quality assessment of the included case-control (n=22) and cohort studies (n=20) using the Newcastle-Ottawa Scale

Quality assessment of the included cohort and case-control studies using the Newcastle-Ottawa Scale. The scores for the three separate parts, consisting of selection, comparability and outcome assessment are displayed next to every article and the maximum score is indicated at the headings.

The three parts were: selection (0 – 4 points), comparability (0 – 2 points), and outcome assessment (0 – 3 points).

Table S3. Quality assessment of the included case series (n=1) using an adjusted Newcastle-Ottawa Scale

This adjusted NOS consists of four parts: selection (0 – 1 point), ascertainment (0 – 2 points), causality (0 – 4 points, of which one was not included for the quality assessment of this study) and reporting (0 – 1 point).

Table S4. Compound database for MetScape 3

Compounds (n=129) included in the pathway analysis of paediatric patients with overweight and obesity, combined with their KEGG ID, HMDB ID and PubChem ID.

Table S5. Pathway analysis using MetaboAnalyst 5.0

Resultant pathway names (n=38) and their match status in childhood obesity. MetaboAnalyst settings imparted hypergeometric test as over-representation analysis method; relative betweennes centrality as node importance measure for topological analysis; pathway library homo sapiens for human studies. In general, small *P*-values and large pathway impact indicate the most relevant pathways. Yet, as pathway

impact and corresponding *P*-value are relative importance measures and thus greatly influenced by the number of imported differential (targeted) metabolites, only the number of hits was addressed as a relevant threshold in appointing the most important pathways altered in obesity.

Fig S1. Visualisation of pathway analysis, using MetaboAnalyst 4.0

Pathway analysis of paediatric patients with overweight and obesity, with the most important pathways (> 5 hits) being: (1) aminoacyl-tRNA biosynthesis, (2) Valine, leucine and isoleucine biosynthesis, (3) Biosynthesis of unsaturated fatty acid, (4) Arginine biosynthesis, (5) Steroid hormone biosynthesis, (6) Pantothenate and CoA biosynthesis, (7) Primary bile acid synthesis, (8) Glycine, serine and threonine metabolism, (9) beta-Alanine metabolism (10) Histidine metabolism, (11) Alanine, aspartate and glutamate metabolism, (12) Steroid biosynthesis, (13) Glyoxylate and dicarboxylate metabolism, (14) Valine, leucine and isoleucine degradation (15) Arginine and proline metabolism, (16) Cysteine and methionine metabolism, (17) Tyrosine metabolism, (18) Galactose metabolism, (19) Purine metabolism, (20) Glutathione metabolism and (21) Linoleic metabolism.

Figure S2. The metabolic network and pathway mapping

Compound-reaction based metabolic network using MetScape 3 with user-inputted compounds (red hexagons), linking metabolites (pink hexagons) and database generated reactions (beige rectangles) as nodes, linking compounds and reactions on the basis of metabolomic data sources (i.e. KEGG and Human Metabolome Database). Putative interactions among the altered metabolites in childhood obesity were visualised with a clear interplay of several pathways. The different shades of red, blue and green colored shapes refer to diverse pathways in lipid, carbohydrate and amino acid metabolism, respectively.

Additional File 2:

Table S1. Database including all data extracted from selected studies

Database concerning altered metabolites in paediatric patients with overweight and obesity in separate excel file, including first author and title, year of publication, continent, country of the study, study

design, sample size, diagnostic criteria, characteristics of the study populations (age, tanner stage and sex), analytical technique, biological matrix studied and quantitative findings, if these were available.

ADDITIONAL FIGURES

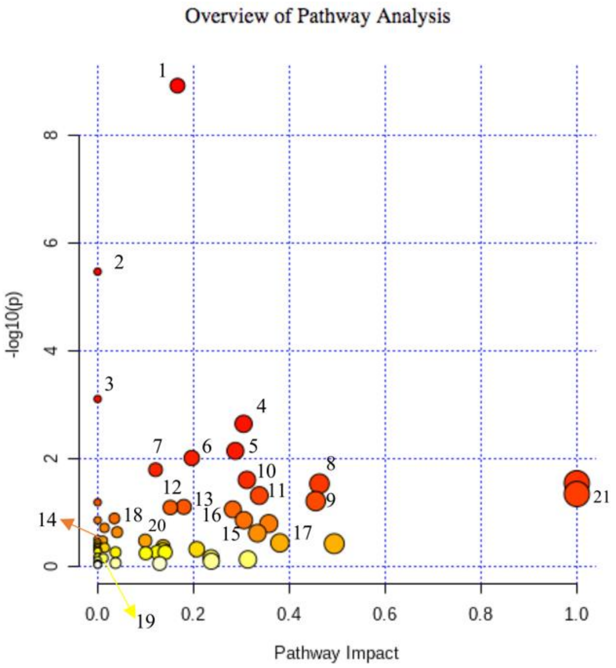

Figure S1. Visualisation of pathway analysis, using MetaboAnalyst 4.0

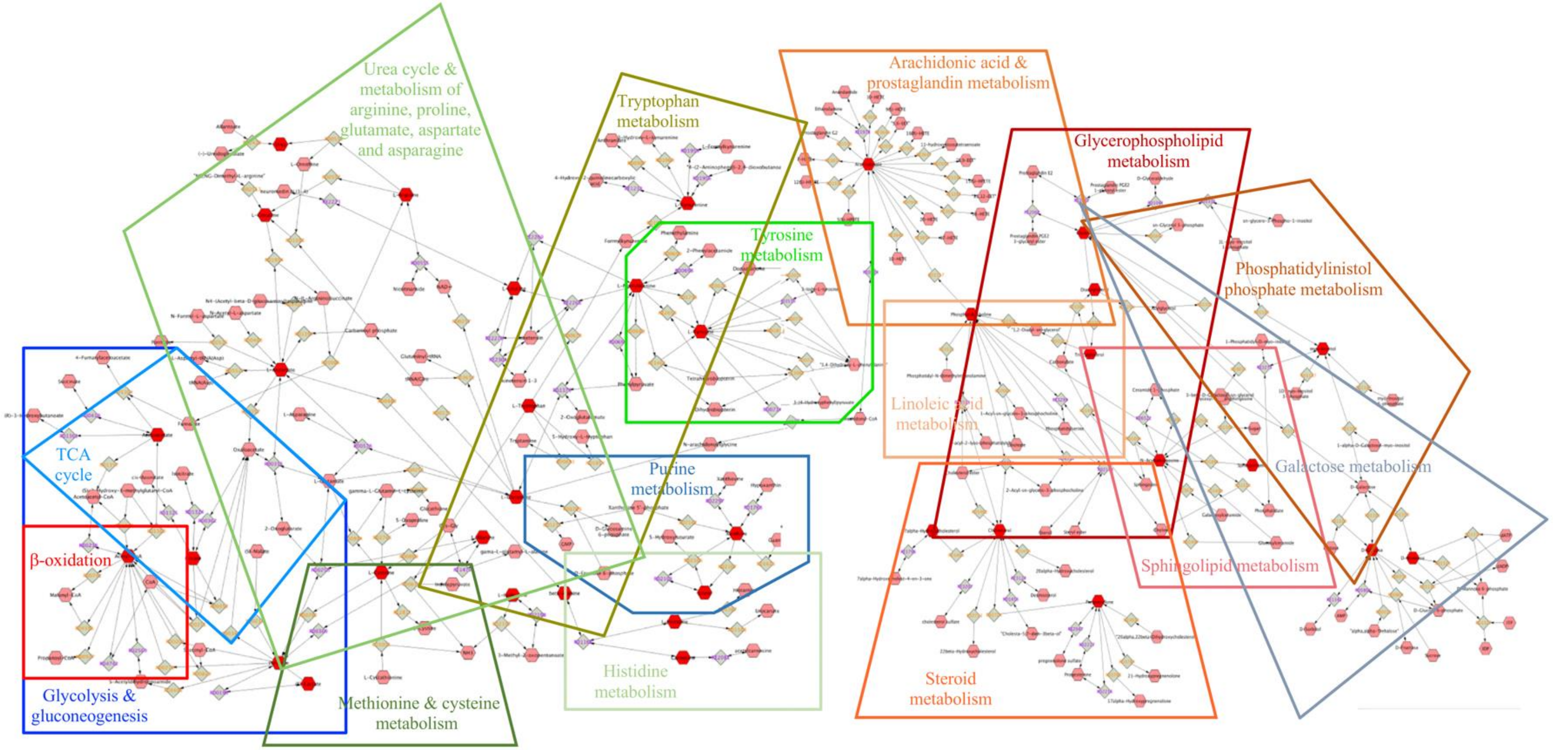

Figure S2. The metabolic network and pathway mapping

ADDITIONAL TABLES

Table S1. Pragmatic database search strategy according to the PICO framework

	FIELD	Search terms
Population	Topic	(‘Child*’ OR ‘Bab*’ OR ‘Boy*’ OR ‘Girl*’ OR ‘Infants’ OR ‘Neonate*’ OR ‘Childhood’ OR ‘Pub*’ OR ‘Pre-pubertal’ OR ‘Prepubertal’ OR ‘Adolescents’)
AND		
Outcome	Topic	(“Type 2 diabetes” OR ‘Pre-diabetes’ OR ‘Prediabetes’ OR “Impaired glucose tolerance” OR ‘Db2’ OR ‘T2D’ OR “Metabolic syndrome” OR ‘MetS’ or ‘Obesity’ OR ‘IR’ OR "Insulin resistance")
AND		
		(‘Metabolomics’ OR ‘Metabolite’ OR ‘Lipid*’)
AND		
		(‘Urine’ OR ‘Plasma’ OR ‘Serum’ OR ‘Blood’ OR ‘Excretion’ OR ‘Hair’ OR ‘*nail’ OR ‘Saliva’ OR ‘Feces’ OR ‘Faeces’)
AND		
Type of study	Topic	(‘Trial’ OR ‘Experiment’ OR ‘Study’ OR ‘Intervention’ OR ‘Cohort’)
AND		
Limitations	Language	English
	Document type	Article
	Year Published/ Publication Period	2015 – 2021 (31/01/2021)

Table S2. Quality assessment according to the Newcastle-Ottawa Scale

Continent	Reference	Study design	Selection (4)	Comparability (2)	Outcome assessment (3)	Total (9)
ASIA	Cho *et al.* 2017	Cohort	4	2	3	9
	Kim *et al.* 2016	Case-control	3	2	2	7
	Lee *et al.* 2018	Cohort	3	2	2	7
	Lee *et al.* 2019	Cohort	3	2	2	7
	Son et al. 2019	Case-control	3	2	3	8
AUSTRALIA	Saner *et al.* 2018	Cohort	2	2	3	7
	~~Ke *et al.* 2017~~	~~Cohort~~	~~1~~	~~2~~	~~2~~	~~5~~
EUROPE	Anjos *et al.* 2019	Case-control	2	2	2	6
	Hosking *et al.* 2019	Cohort	4	2	2	8
	Lau *et al.* 2018	Cohort	4	2	2	8
	Mangge *et al.* 2015	Case-control	3	2	2	7
	Martos-Moreno *et al.* 2017	Case-control	3	2	3	8
	Mastrangelo *et al.* 2016	Case-control	4	2	2	8
	Reinehr *et al.* 2015	Case-control	3	2	3	8
	Rocha *et al.* 2018	Case-control	3	2	3	8
	Troisi *et al.* 2017	Case-control	3	2	2	7
	Troisi *et al.* 2019	Case-control	3	2	2	7
	Valle *et al.* 2015	Case-control	3	2	2	7
	Wahl *et al.* 2012	Case-control	3	1	2	6
	Wijnant *et al.* 2020	Case-control	2	2	2	6
	Zhang *et al.* 2019	Cohort	2	2	3	7
AMERICA	Chavira-Suarez *et al.* 2020	Case-control	4	2	3	8
	Flannagan *et al.* 2018	Cohort	3	2	2	7
	Goffredo *et al.* 2017	Case-control	3	2	3	8
	Higgins *et al.* 2020	Cohort	3	2	3	8
	Mauras *et al.* 2015	Case-control	3	2	3	8
	Perng *et al.* 2018	Cohort	4	2	3	9
	Perng *et al.* 2020	Case-control	2	2	3	7
	Short *et al.* 2019	Case-control	3	2	3	8

~~Sorrow *et al.* 2019~~	~~Case-control~~	~~1~~	~~2~~	~~2~~	~~5~~
~~Syme *et al.* 2019~~	~~Cohort~~	~~1~~	~~2~~	~~1~~	~~4~~
Trico *et al.* 2017	Cohort	4	2	3	9
Trico *et al.* 2019	Case-control	3	2	3	8
Aristizabal *et al.* 2017	Case-control	3	2	2	7
Bermudez-Cardona and Velasquez-Rodriguez 2016	Case-control	3	2	3	8
Butte *et al.* 2015	Cohort	4	2	2	8
Cormack *et al.* 2013	Cohort	2	2	2	6
Farook *et al.* 2015	Case-control	4	2	2	8
Moran-Ramos *et al.* 2017	Cohort	4	2	2	8
Perng *et al.* 2017	Cohort	3	2	3	8
Perng *et al.* 2019	Cohort	3	2	3	8
Newbern *et al.* 2014	Cohort	3	2	3	8

Table S3. Quality assessment of the included case series (n=1) using an adjusted Newcastle-Ottawa Scale.

Continent	Reference	Selection (1)	Ascertainment (2)	Causality (3)	Reporting (1)	Total (7)
ASIA	Suzuki et al. 2019	0	2	2	1	5

Table S4. Compound database for MetScape 3

	Component	KEGG ID	HMDB ID
1	1-methylhistidine	C01152	
2	13-OxoODE	C14765	
3	16a-Hydroxyestrone	C05300	
4	17-OH-pregnenolone	C18038	
5	2-aminoadipic acid	C00956	
	2-hydroxyacetaminophen sulfate		HMDB0062547
6	2-ketobutyric acid	C00109	
7	2-methoxy-estradiol	C05302	
	2-methoxyacetaminophen glucuronide		HMDB0240215
	2-Methylbutyroylcarnitine		HMDB0000378
8	2-oxovaleric acid	C06255	
	2-palmitoylglycerol		HMDB0011533
	2-piperidinone		HMDB0011749
9	24S-hydroxycholesterol	C13550	
	3-(N-acetyl-L-cystein-S-yl) acetaminophen		HMDB0240217
10	3-hydroxybutyrate	C01089	
11	3-Hydroxyquinine	C07344	
12	3-methyl-2-oxovaleric acid	C03465	
13	3,4-dihydroxyphenylalanine	C00355	
14	3beta-7alpha-dihydroxy-5-cholestenoic acid	C17335	
15	3beta-Hydroxy-5-cholestenoic acid	C17333	
	4-androsten-3beta,17beta-diol disulfate		HMDB0240313
	4-deoxyerythronic acid		HMDB0000498
16	4-hydroxyproline	C01157	
17	4-methyl-2-oxopentanoic acid	C00233	
18	5-oxoproline	C01879	
19	7alpha-hydroxy-3-oxo-4-cholestenoic acid	C17337	
20	7alpha-hydroxycholesterol	C03594	
	7beta-hydroxycholesterol		HMDB0006119
21	9-OxoODE	C14766	
22	Acetic acid	C00033	
23	Acetoacetic acid	C00164	
24	L-acetylcarnitine	C02571	
25	L-alanine	C01401	
	L-alloisoleucine		HMDB0000557
26	alpha-aminoisobutric acid	C03665	
27	alpha-hydroxybutyric acid	C05984	
28	alpha-linolenic acid 18:3(n-3)	C06427	
29	Androstenedione	C00280	
30	Androsterone	C00523	
	Androsterone sulfate		HMDB0002759
31	Arachidonic acid 20:4(n-6)	C00219	
32	L-arginine	C00062	
33	L-asparagine	C00152	
34	L-Aspartic acid	C00049	
35	Behenic acid	C08281	
36	beta-alanine	C00099	
37	Bradykinin	C00306	
38	Butyryl-L-carnitine	C02862	
39	Campesterol	C01789	
40	Caprylic acid	C06423	
41	Carnosine	C00386	
42	Chenodeoxycholic acid	C02528	
43	Cholesterol	C00187	
44	Cholesteryl arachidonate	C02530	
45	Citrate/Citric acid	C00158	
46	Citrulline	C00327	

47	Cortisone	C00762	
48	Cystathionine	C00542	
49	L-Cysteïne	C00097	
50	D-Glucose	C00031	
51	D-Maltose	C00208	
52	D-Mannose	C00159	
	Decanoylcarnitine		HMDB0000651
	Decenoylcarnitine		HMDB0013205
53	Dehydroepiandrosterone	C01227	
54	Dehydroepiandrosterone sulfate	C04555	
55	DAG 16:0/16:0	C00165	
56	Dihomo-gamma-linolenic 20:3(n-6)	C03242	
57	Docosahexaenoic acid 22:6(n-3)	C06429	
	Docosapentaenoic acid 20:5(n-3)		HMDB0001976
	Dodecanoylcarnitine		HMDB0002250
58	Dodecenedioic acid	C16308	
	Dodecenoylcarnitine		HMDB0013326
59	Dopamine	C03758	
60	Eicosapentaenoic acid 20:5(n-3)	C06428	
	Epiandrosterone sulfate		HMDB0062657
61	Estradiol	C00951	
62	DL-Glutamate	C00302	
63	L-glutamine	C00064	
64	Glycerol	C00116	
65	Glycine	C00037	
66	Glycodeoxycholate	C05464	
	Hexadecenoylcarnitine		HMDB0013207
	Hexanoylcarnitine		HMDB0000756
67	L-Histidine	C00135	
	Hydroxyisovalerylcarnitine		HMDB0061189
	Hydroxypropionylcarnitine		HMDB0013125
	Hydroxyvalerylcarnitine		HMDB0013132
	Indole-3-propionic acid		HMDB0002302
68	L-Isoleucine	C00407	
69	Isovalerylcarnitine	C20826	
70	Kynurenic acid	C01717	
71	L-Carnitine	C00318	
72	L-Kynurenine	C01718	
	L-Thyronine		HMDB0000667
73	(S)-Lactate	C00186	
74	Lanosterol	C01724	
75	Lathosterol	C01189	
76	Lauric acid	C02679	
77	L-Leucine	C00123	HMDB0000687
78	Linoleic-18:2(n-6)	C01595	
	Linoleylcarnitine (C18:2)		HMDB0006469
79	Lithocholic acid	C03990	
	LysoPC 20:4		
	LysoPC 18:1		HMDB0002815
	LysoPC 18:2		HMDB0010386
80	L-Lysine	C00047	
81	LysoPC 14:1	C04230	
82	LysoPC 16:0	C04230	
83	LysoPC 14:0	C04230	
84	LysoPC 16:1	C04230	
85	LysoPC 17:0	C04230	
	LysoPC 18:1		HMDB0002815
	LysoPC 18:2		HMDB0010386
	Malonylcarnitine		HMDB0002095
	Malvidin 3-(6-acetyl glucoside)		HMDB0038008

86	L-Methionine	C00073	
	Methylglutarylcarnitine C6DC		
87	Myo-inositol	C00137	
88	Myristic acid	C06424	
89	N-acetyl galactosamine	C05021	
	N-acetylglycine		HMDB0000532
90	N,N-dimethylarginine	C03626	
91	Naringenin	C00509	
	Nonaoylcarnitine		HMDB0013288
92	Octanoylcarnitine	C02838	
93	Octenoylcarnitine	C02838	
94	Oleic acid	C00712	
95	Ornithine	C01602	
	P-acetamidophenyl glucuronide		HMDB0010316
96	Palmitic acid	C00249	
97	Palmitoleic acid	C08362	
98	Palmitoyl-linoleoyl-glycerol (16:0/18:2)	C00165	
99	L-palmitoylcarnitine	C02990	
100	Panthothenate/coenzym A	C00010	
	PC 44:10		HMDB0008713
	PC 28:1, PC(14:1/14:0)		HMDB0007867
	PC 30:2, PC(14:1(9Z)/16:1(9Z))		HMDB0007998
	PC 32:2 (16:1/16:1)		HMDB0013411
	PC 34:1, PC(16:0/18:1(11Z))		HMDB0007971
	PC 34:2, PC(18:1(9Z)/16:1(9Z))		HMDB0013413
	PC 34:4, PC(18:4(6Z,9Z,12Z,15Z)/16:0)		HMDB0008258
101	PC 36:1, PC(14:0/22:1(13Z))	C00157	
	PC 38:0, PC(20:0/18:0)		HMDB0008043
	PC 38:6, PC(22:6(4Z,7Z,10Z,13Z,16Z,19Z)/P-16:0)		HMDB0011229
	PC 40:6, PC(22:5(4Z,7Z,10Z,13Z,16Z)/P-18:1(9Z))		HMDB0011326
	PC ae C34:1, PC(O-16:1(9Z)/18:0)		HMDB0013426
	PC ae C34:2, PC(O-16:1(9Z)/18:2)		HMDB0011151
	PC ae C34:3, PC(O-16:1(9Z)/18:2(9Z,12Z))		HMDB0013413
	PC ae C36:2, PC(O-18:0/18:2(9Z,12Z))		HMDB0013418
	PC ae C36:3, PC(O-18:1/18:2(9Z,12Z))		HMDB0013429
	PC ae C38:2, PC(O-18:1(9Z)/20:1(11Z))		HMDB0013431
	PC ae C38:5, PC(O-18:1(9Z)/20:4(8Z,11Z,14Z,17Z))		HMDB0013432
	PC ae C38:6, PC(O-16:0/22:6(4Z,7Z,10Z,13Z,16Z,19Z))		HMDB0013409
	PC ae C44:4, PC(O-22:1(13Z)/22:3(10Z,13Z,16Z))		HMDB0013453
	PC ae C44:5, PC(O-22:2(13Z,16Z)/22:3(10Z,13Z,16Z))		HMDB0013456
	PE 30:3		
	PE 38:9		HMDB0009203
	PE 42:9		HMDB0009406
102	L-Phenylalanine	C00079	HMDB0000159
103	Phosphatidylethanolamine	C00346	
	PI 34:1, PI(16:0/18:1(9Z))		HMDB0009799
	PI 34:2, PI(18:1(9Z)/16:1(9Z))		HMDB0009799
	PI 36:1		HMDB0240667
	PI 36:2, PI(18:2(9Z,12Z)/18:0)		HMDB0009807
	PI 36:3, PI(18:2(9Z,12Z)/18:1(9Z))		HMDB0009840
	PI 36:4, PI(18:1(9Z)/18:3(9Z,12Z,15Z))		HMDB0009840
	PI 38:3, PI(20:1(11Z)/18:2(9Z,12Z))		HMDB0009872
	PI 38:4, PI(20:4(5Z,8Z,11Z,14Z)/18:0)		HMDB0009815
	PI 38:5, PI(16:2(9Z,12Z)/22:3(10Z,13Z,16Z))		HMDB0009804

104	Piperidine	C01746	
105	Pregnenolone	C01953	
106	Pregnenolone sulfate	C18044	
107	Proline	C00148	
108	Propionylcarnitine	C03017	
	PS 30:2, PS(16:1(9Z)/14:1(9Z))		HMDB0012333
109	Putrescine	C00134	
110	Pyroglutamic acid	C02237	
111	Pyruvic acid	C00022	
112	L-Serine	C00065	
113	SM (OH) 14:1, SM(d18:0/14:1(9Z)(OH))	C00550	
114	SM (OH) 16:1, SM(d18:0/16:1(9Z)(OH))	C00550	
115	SM (OH) 22:1, SM(d18:0/22:1(13Z)(OH))	C00550	
	SM 16:0		HMDB0010169
116	Stearic acid	C01530	
	Stearoylcarnitine		HMDB0000848
117	Stigmasterol	C05442	
118	Taurodeoxycholic acid	C05463	
119	Taurolithocholate 3-sulfate	C03642	
120	Testosterone	C00535	
121	L-Threonine	C00188	
122	Thymine	C00178	
123	Trimethylamine-N-oxide	C00565	
124	L-Tryptophan	C00078	
125	L-Tyrosine	C00082	
126	Urea	C00086	
127	Uric acid	C00366	
	Valerylcarnitine		HMDB0013128
128	L-Valine	C00183	
129	Xanthine	C00385	

Table S5. Pathway analysis using MetaboAnalyst 5.0

**Pathway**	**Total**	**Hits**	**Corrected *p*-value**	**Impact**
Aminoacyl-tRNA biosynthesis	48	18	1.42E-09	0.17
Valine, leucine and isoleucine biosynthesis	8	6	3.65E-06	0.01
Biosynthesis of unsaturated fatty acids	36	9	8.51E-04	0.01
Arginine biosynthesis	14	5	2.40E-03	0.30
Steroid hormone biosynthesis	85	12	7.90E-03	0.29
Pantothenate and CoA biosynthesis	19	5	1.03E-02	0.12
Primary bile acid biosynthesis	46	8	1.72E-02	0.12
Histidine metabolism	16	4	2.60E-02	0.31
Phenylalanine, tyrosine and tryptophan biosynthesis	4	2	1.53E-02	1.00
Glycine, serine and threonine metabolism	33	6	1.51E-02	0.46
Linoleic acid metabolism	5	2	4.70E-02	1.00
Alanine, aspartate and glutamate metabolism	28	5	5.10E-02	0.34
beta-Alanine metabolism	21	4	6.43 E-02	0.46
D-Glutamine and D-glutamate metabolism	6	2	6.72E-02	0.00
Glyoxylate and dicarboxylate metabolism	32	5	8.30E-02	0.18
Steroid biosynthesis	42	6	8.53E-02	0.15
Cysteine and methionine metabolism	33	5	9.20E-02	0.28
Galactose metabolism	27	4	1.35E-01	0.03
Arginine and proline metabolism	38	5	1.43E-01	0.31
Glutathione metabolism	28	4	1.49E-01	0.11
Phenylalanine metabolism	10	2	1.64E-01	0.36
Sphingolipid metabolism	21	3	1.97E-01	0.01
Propanoate metabolism	23	3	8.64E-01	0.04
alpha-Linolenic acid metabolism	13	2	2.47E-01	0.33
Valine, leucine and isoleucine degradation	40	4	3.39E-01	0.01
Tyrosine metabolism	42	4	3.73E-01	0.38
Starch and sucrose metabolism	18	2	3.86E-01	0.49
Citrate cycle (TCA cycle)	20	2	4.39E-01	0.134
Fatty acid biosynthesis	47	4	4.59E-01	0.01
Pyruvate metabolism	22	2	4.90E-01	0.21
Glycerophospholipid metabolism	36	3	5.01E-01	0.14
Pyrimidine metabolism	39	3	5.57E-01	0.04
Fatty acid degradation	39	3	5.57E-01	0.12
Lysine degradation	25	2	5.60E-01	0.14
Glycolysis / Gluconeogenesis	26	2	5.82E-01	0.10
Purine metabolism	65	4	7.19E-01	0.01
Arachidonic acid metabolism	36	2	7.57E-01	0.31
Tryptophan metabolism	41	2	8.18E-01	0.24
